# Genomic prediction using models with dominance and imprinting effects for backfat thickness and average daily gain in Danish Duroc pigs

**DOI:** 10.1186/s12711-016-0245-6

**Published:** 2016-09-13

**Authors:** Xiangyu Guo, Ole Fredslund Christensen, Tage Ostersen, Yachun Wang, Mogens Sandø Lund, Guosheng Su

**Affiliations:** 1 Center for Quantitative Genetics and Genomics, Department of Molecular Biology and Genetics, Aarhus University, 8830 Tjele, Denmark; 2 Danish Pig Research Centre, SEGES P/S, 1609 Copenhagen, Denmark; 3 College of Animal Science and Technology, China Agricultural University, Beijing, 100193 People’s Republic of China

## Abstract

**Background:**

Dominance and imprinting genetic effects have been shown to contribute to genetic variance for certain traits but are usually ignored in genomic prediction of complex traits in livestock. The objectives of this study were to estimate variances of additive, dominance and imprinting genetic effects and to evaluate predictions of genetic merit based on genomic data for average daily gain (DG) and backfat thickness (BF) in Danish Duroc pigs.

**Methods:**

Corrected phenotypes of 8113 genotyped pigs from breeding and multiplier herds were used. Four Bayesian mixture models that differed in the type of genetic effects included: (A) additive genetic effects, (AD) additive and dominance genetic effects, (AI) additive and imprinting genetic effects, and (ADI) additive, dominance and imprinting genetic effects were compared using Bayes factors. The ability of the models to predict genetic merit was compared with regard to prediction reliability and bias.

**Results:**

Based on model ADI, narrow-sense heritabilities of 0.18 and 0.31 were estimated for DG and BF, respectively. Dominance and imprinting genetic effects accounted for 4.0 to 4.6 and 1.3 to 1.4 % of phenotypic variance, respectively, which were statistically significant. Across the four models, reliabilities of the predicted total genetic values (GTV, sum of all genetic effects) ranged from 16.1 (AI) to 18.4 % (AD) for DG and from 30.1 (AI) to 31.4 % (ADI) for BF. The least biased predictions of GTV were obtained with model AD, with regression coefficients of corrected phenotypes on GTV equal to 0.824 (DG) and 0.738 (BF). Reliabilities of genomic estimated breeding values (GBV, additive genetic effects) did not differ significantly among models for DG (between 16.5 and 16.7 %); however, for BF, model AD provided a significantly higher reliability (31.3 %) than model A (30.7 %). The least biased predictions of GBV were obtained with model AD with regression coefficients of 0.872 for DG and 0.764 for BF.

**Conclusions:**

Dominance and genomic imprinting effects contribute significantly to the genetic variation of BF and DG in Danish Duroc pigs. Genomic prediction models that include dominance genetic effects can improve accuracy and reduce bias of genomic predictions of genetic merit.

## Background

Dominance is an interaction effect between the two alleles at the same locus [1]. Dominance effects were reported to contribute significantly to the genetic variation of reproduction and production traits in pigs, with dominance variance accounting for 2.2 to 10.3 % of the phenotypic variance [2]. Imprinting is the result of an epigenetic process in which some genes are expressed in a parent-of-origin-specific manner, which is caused by DNA methylation and histone modifications [3]. An imprinted gene is expressed at a lower level than the copy from the other parent. At each generation, imprinting of genes is established de novo during gametogenesis [4]. In pigs, quantitative traits such as carcass composition, growth, and reproduction traits have been found to be influenced by imprinting effects [4–8]. de Vries et al. [9] reported that imprinting accounted for approximately 5 % of the phenotypic variance of backfat thickness and up to 4 % for average daily gain in Landrace and Yorkshire pigs. More specifically, the *insulin*-*like growth factor 2* (*IGF2*) gene in pigs is expressed only from the paternal allele (i.e., maternal imprinting) [10]. A polymorphism in this gene affects muscle mass and fat deposition [10]. Although dominance and imprinting effects contribute to the variation in backfat and daily gain [2, 9], these sources of variation are usually not included in models for genetic evaluation.

With the development of high-throughput genotyping technologies, it is possible to predict breeding values and carry out selection by using information from genome-wide single-nucleotide polymorphisms (SNPs), which is known as genomic selection [11]. Instead of detecting quantitative trait loci (QTL) or chromosomal regions that significantly affect traits of interest, as in genome-wide association studies [12], genomic selection focuses on the prediction of additive or total genetic effects [12]. Recently, some studies have compared genomic predictions that were obtained by using models with or without dominance and imprinting effects [13, 14]. However, information on the impact of dominance and imprinting effects on genomic selection is scarce. Therefore, the objectives of our study were to estimate the genetic parameters of additive, dominance and imprinting effects and assess their impact on the accuracy and bias of predictions of genetic merit for average daily gain and backfat thickness in Danish Duroc pigs, using genomic data.

## Methods

### Data

The traits included in the analyses were average daily gain (DG = weight gain/days) from 30 to 100 kg and ultrasound measured backfat thickness (BF) at approximately 100 kg on Danish Duroc pigs. The data were collected from breeding and multiplier herds and were supplied by SEGES P/S, the Danish Pig Research Centre. Because non-genetic effects are more precisely estimated from a large dataset than from a small subset of genotyped individuals, we used corrected phenotypic values $\left( {y_{c} } \right)$ of BF and DG instead of original observations as response variables for genotyped individuals. Adjusted phenotypes were computed as the original observations for DG or BF adjusted for non-genetic effects of the combination of herd, stable, year and season, of sex (female or male) and pen, using a single-trait model. For DG, start weight was also adjusted for measurements of DG (989 ± 125 g) and BF (8.56 ± 1.71 mm) on 421,092 individuals born between 1992 and 2014 were used to estimate the adjustments.

In total, 12,206 pigs were genotyped using the Illumina PorcineSNP60 BeadChip or the 8.5 K GGP-Porcine LD Illumina Bead SNP Chip (Illumina, San Diego). SNP data for the 990 animals that were genotyped with the 8.5 K GGP-Porcine LD Illumina Bead SNP Chip were imputed to the 60 K chip by using Beagle version 3.3.1 [15]. In total, 33,004 SNPs across the 18 pig autosomes met the following requirements: (1) each SNP had a minor allele frequency higher than 0.01, (2) a call rate score greater than 0.9, (3) no strong deviation from Hardy–Weinberg equilibrium (*P* > 10^−7^ for a Chi square test), and (4) a known position on the porcine Build 10.2 assembly [16]. Moreover, for each animal and SNP-genotype combination, if the GenCall score was less than 0.6, genotypes were defined as missing and animals for which the call rate was less than 0.8 were excluded from the analysis.

To distinguish the parental origin of the chromosomes, the SNP genotype data were phased by using FImpute version 2.2 [17], taking pedigree information into account. Only animals with at least one genotyped parent were included in the subsequent analyses. Consequently, 8113 pigs (6418 boars and 1695 sows) born between 1998 and 2014 and originating from 818 sires and 4666 dams were included in the analysis. The data included 1953 full-sib families, 517 paternal half-sib families, and 196 maternal half-sib families with two or more individuals and average sizes of 2.44, 6.01 and 2.05, respectively.

### Statistical models

Previous studies on the inclusion of imprinting effects in a statistical model used various definitions of additive genetic variance. Some studies defined the additive genetic variance as the variance of parent-of-origin independent additive genetic effects and the imprinting variance as the variance of parent-of-origin dependent additive genetic effects [13, 18]. Other studies defined the total additive genetic variance as the sum of parent-of-origin independent and parent-of-origin dependent additive genetic variances [19, 20]. Consequently, the former definition resulted in the narrow-sense heritability being equal to the proportion of parent-of-origin independent additive genetic variance relative to phenotypic variance [9, 13], while with the latter definition, it was equal to the proportion of the sum of parent-of-origin independent and parent-of-origin dependent additive genetic variances relative to phenotypic variance [19]. For ease of presentation and comparison between models, we used the former definition to determine narrow-sense heritability. Broad**-**sense heritability was defined as the proportion of the total genetic variance relative to phenotypic variance [13, 14].

The following four Bayesian mixture models were used to describe the corrected phenotypic values [21]. The full model assumed that the genetic effect of a phenotype was decomposed into additive, dominance and imprinting effects, and these were estimated simultaneously:

ADI $${\mathbf{y}}_{{\mathbf{c}}} = {\mathbf{Xb}} + {\mathbf{M}}_{{\mathbf{a}}} {\mathbf{q}}_{{\mathbf{a}}} + {\mathbf{M}}_{{\mathbf{d}}} {\mathbf{q}}_{{\mathbf{d}}} + {\mathbf{M}}_{{\mathbf{i}}} {\mathbf{q}}_{{\mathbf{i}}} + {\mathbf{Z}}_{{\mathbf{l}}} {\mathbf{l}} + {\mathbf{e}},$$

where ${\mathbf{y}}_{{\mathbf{c}}}$ is the vector of corrected phenotypic values of DG or BF; **b** is the vector of the fixed effect of birth year; **X** is the incidence matrix associating **b** with ${\mathbf{y}}_{{\mathbf{c}}} ;\,{\mathbf{q}}_{{\mathbf{a}}} ,\,{\mathbf{q}}_{{\mathbf{d}}}$ and ${\mathbf{q}}_{{\mathbf{i}}}$ are vectors of additive, dominance and imprinting effects of SNPs, respectively; ${\mathbf{M}}_{{\mathbf{a}}} ,\,{\mathbf{M}}_{{\mathbf{d}}}$ and ${\mathbf{M}}_{{\mathbf{i}}}$ are ${\text{n}} \times {\text{N}}_{\text{snp}}$ matrices (n is the number of genotyped individuals, and ${\text{N}}_{\text{snp}}$ is the number of SNPs), defined below; **l** is the vector of random litter effects; ${\mathbf{Z}}_{{\mathbf{l}}}$ is the incidence matrix associating **l** with ${\mathbf{y}}_{{\mathbf{c}}}$; and **e** is the vector of residuals.

We assumed that additive, dominance and imprinting effects were independent from each other and that the prior distribution of each of these effects was a mixture of four normal distributions:

$${\mathbf{q}}\,\sim\,\pi_{1} {\text{N}}\left( {{\bf 0},\sigma_{{\pi_{1} }}^{2} } \right) + \pi_{2} {\text{N}}\left( {{\bf 0},\sigma_{{\pi_{2} }}^{2} } \right) + \pi_{3} N\left( {{\bf 0},\sigma_{{\pi_{3} }}^{2} } \right) + \pi_{4} {\text{N}}\left( {{\bf 0},\sigma_{{\pi_{4} }}^{2} } \right),$$

where **q** is ${\mathbf{q}}_{{\mathbf{a}}} ,\,{\mathbf{q}}_{{\mathbf{d}}}$ or ${\mathbf{q}}_{{\mathbf{i}}}$. Mixing proportions in this distribution were assumed known and set to $\pi_{1}$ = 0.889, $\pi_{2}$ = 0.1, $\pi_{3}$ = 0.01, and $\pi_{4}$ = 0.001 [21]; the variances were assumed to be model parameters and were estimated by assuming flat prior distributions under the constraint $\sigma_{{\pi_{1} }}^{2} < \sigma_{{\pi_{2} }}^{2} < \sigma_{{\pi_{3} }}^{2} < \sigma_{{\pi_{4} }}^{2} .$ Vectors of random litter effects and residuals were assumed to follow a normal distribution, i.e., ${\mathbf{l}}\,\sim\,N\left( {0,{\mathbf{I}}\sigma_{l}^{2} } \right)$ and ${\mathbf{e}}\,\sim\,N\left( {0,{\mathbf{I}}\sigma_{e}^{2} } \right),$ where $\sigma_{l}^{2}$ and $\sigma_{e}^{2}$ are the variances of litter effects and residuals, which were assumed to follow flat prior distributions, and **I** is an identity matrix.

Considering an autosomal biallelic locus with alleles ${\text{A}}_{1}$ and ${\text{A}}_{2}$ with frequencies 1 − *p* and *p*, respectively, in the population, the elements of ${\mathbf{M}}_{{\mathbf{a}}} ,\,{\mathbf{M}}_{{\mathbf{d}}}$, and ${\mathbf{M}}_{{\mathbf{i}}}$ for the ith individual at the jth SNP were calculated as follows [14], depending on SNP genotype:

$$\begin{aligned} {\mathbf{M}}_{{{\mathbf{a}}ij}} & = \left\{ {\begin{array}{*{20}l} { - 2p_{j} } \hfill &\quad {{\text{A}}_{1} {\text{A}}_{1} } \hfill \\ {1 - 2p_{j} } \hfill &\quad {{\text{A}}_{1} {\text{A}}_{2} \left( {{\text{A}}_{2} {\text{A}}_{1} } \right)} \hfill \\ {2 - 2p_{j} } \hfill &\quad {{\text{A}}_{2} {\text{A}}_{2} } \hfill \\ \end{array} } \right., \\ {\mathbf{M}}_{{{\mathbf{d}}ij}} & = \left\{ {\begin{array}{*{20}l} { - 2p_{j}^{2} } \hfill & {{\text{A}}_{1} {\text{A}}_{1} } \hfill \\ {2p_{j} \left( {1 - p_{j} } \right)} \hfill & {{\text{A}}_{1} {\text{A}}_{2} \left( {{\text{A}}_{2} {\text{A}}_{1} } \right)} \hfill \\ { - 2\left( {1 - p_{j} } \right)^{2} } \hfill & {{\text{A}}_{2} {\text{A}}_{2} } \hfill \\ \end{array} } \right., \\ {\mathbf{M}}_{{{\mathbf{i}}ij}} & = \left\{ {\begin{array}{*{20}l} 0 \hfill & {{\text{A}}_{1} {\text{A}}_{1} } \hfill \\ 1 \hfill & {{\text{A}}_{1} {\text{A}}_{2} } \hfill \\ { - 1} \hfill & {{\text{A}}_{2} {\text{A}}_{1} } \hfill \\ 0 \hfill & {{\text{A}}_{2} {\text{A}}_{2} } \hfill \\ \end{array} } \right.. \\ \end{aligned}$$

The data were also analyzed using three reduced models, which excluded either imprinting or dominance effects, or both, from the full model as follows:

AD $${\mathbf{y}}_{{\mathbf{c}}} = {\mathbf{Xb}} + {\mathbf{M}}_{{\mathbf{a}}} {\mathbf{q}}_{{\mathbf{a}}} + {\mathbf{M}}_{{\mathbf{d}}} {\mathbf{q}}_{{\mathbf{d}}} + {\mathbf{Z}}_{{\mathbf{l}}} {\mathbf{l}} + {\mathbf{e}},$$

AI $${\mathbf{y}}_{{\mathbf{c}}} = {\mathbf{Xb}} + {\mathbf{M}}_{{\mathbf{a}}} {\mathbf{q}}_{{\mathbf{a}}} + {\mathbf{M}}_{{\mathbf{i}}} {\mathbf{q}}_{{\mathbf{i}}} + {\mathbf{Z}}_{{\mathbf{l}}} {\mathbf{l}} + {\mathbf{e}},$$

A $${\mathbf{y}}_{{\mathbf{c}}} = {\mathbf{Xb}} + {\mathbf{M}}_{{\mathbf{a}}} {\mathbf{q}}_{{\mathbf{a}}} + {\mathbf{Z}}_{{\mathbf{l}}} {\mathbf{l}} + {\mathbf{e}}.$$

The Bayesian analyses were performed using the BayZ package [22] by applying Markov chain Monte Carlo (MCMC) methods. For each analysis, a single Markov chain was conducted with a total length of 50,000 and the first 20,000 samples discarded as burn-in. Convergence and mixing of the chain was assessed by the MCMC error, which was computed using the package ‘coda’ in R [23]; a time-series standard error (SE) <5 % was used to indicate that the MCMC mixed well. Posterior means of the MCMC samples were used as estimates of model parameters. Variance components were computed based on the mixing proportions and variances of four distributions. For example, the additive genetic variance was calculated as:

$$\hat{\sigma }_{a}^{2} = \mathop \sum \limits_{j = 1}^{{{\text{N}}_{\text{snp}} }} 2p_{j} \left( {1 - p_{j} } \right)\mathop \sum \limits_{i = 1}^{4} \pi_{i} \sigma_{{a\pi_{i} }}^{2} ,$$

where $\sigma_{{a\pi_{i} }}^{2}$ is the variance of distribution *i* for additive genetic effects [24]. Significance of the variance components for dominance and imprinting effects in the non-additive models compared to the basic additive model was evaluated by using pseudo Bayes factors (K) [25, 26]. Positive evidence for non-zero variance was declared if twice the natural logarithm of K, i.e., $\left( {2{\text{lnK}}} \right),$ between the alternative model and the additive model ranged from 2 to 6, and very strong evidence was declared if 2lnK ranged from 6 to 10 [27, 28].

### Validation of genomic predictions

The birth date of April 1, 2013, was chosen as the cutoff date to divide the dataset into training and validation datasets of 6250 and 1863 pigs, respectively. Predictive abilities (with respect to reliability and bias) of all models were evaluated by comparing the genomic predictions and $y_{c}$ of individuals in the validation dataset. Reliability of the predictions was evaluated based on the square of the correlation between the predicted genetic values and $y_{c}$ divided by the heritability of $y_{c}$ [1]. Both predictions of additive genetic effects (GBV, genomic estimated breeding value) and total genetic values (GTV, defined as the sum of the genetic effects in the model) were evaluated. The reliability of the predicted GBV was calculated as the squared correlation between the predicted GBV and $y_{c} ,$ divided by the heritability of $y_{c} ,$ i.e., $r^{2} = \frac{{cor^{2} \left( {{\text{GBV}}, y_{c} } \right)}}{{h_{{y_{c} }}^{2} }}$ [1].

Similarly, reliability of the predicted GTV was calculated as:

$$r^{2} = \frac{{cor^{2} \left( {{\text{GTV}}, y_{c} } \right)}}{{h_{{y_{c} }}^{2} }}.$$

Heritability of $y_{c} ,$ i.e., $h_{{y_{c} }}^{2} ,$ was defined as: $h_{{y_{c} }}^{2} = \frac{{\sigma_{a}^{2} }}{{\sigma_{a}^{2} + \sigma_{l}^{2} + \sigma_{e}^{2} }}$ and was used for both GBV and GTV because the heritability of $y_{c}$ was obtained from a traditional pedigree-based model, which considered only the additive genetic effect; thus, the broad-sense heritability was not available. However, because heritability was only used to scale correlation coefficients and was constant for all models, it did not affect the comparison between models. The Hotelling–Williams t test [29, 30] was performed to determine whether reliabilities obtained from models that included dominance and/or imprinting effects were significantly different from those obtained with the basic additive model (A).

Bias of genomic predictions was assessed using the regression of $y_{c}$ on GBV or GTV. A condition for unbiased predictions is that the regression coefficient is 1 and the intercept is 0 [1].

## Results

### Estimates of genetic parameters

Estimated variance components and their proportions of phenotypic variance are in Table 1. The full model ADI, which included additive, dominance and imprinting effects, resulted in the smallest additive genetic, litter and residual variances, while model A, which only considered additive genetic effects, provided the largest estimates for these variances; models AD and AI had estimates for these variances that were intermediate to those of models ADI and A. These results indicate that when dominance and imprinting effects are excluded from a model, the variance due to these effects was assigned to other variance components (i.e., additive genetic variance, variances of litter effect and residual) in the model. Estimates of narrow sense heritability ranged from 17.6 to 19.6 % for DG and from 30.5 to 31.7 % for BF.

**Table 1:** Estimates of variance components from four models of analysis and their proportions relative to the phenotypic variance

Trait	Parameter^a^	ADI	AD	AI	A
DG	$\sigma_{l}^{2}$	390 ± 81	397 ± 78	400 ± 84	424 ± 84
	$\sigma_{a}^{2}$	803 ± 78	828 ± 74	891 ± 74	896 ± 74
	$\sigma_{d}^{2}$	208 ± 46	225 ± 52		
	$\sigma_{i}^{2}$	66 ± 23		88 ± 24	
	$\sigma_{e}^{2}$	3093 ± 97	3138 ± 94	3208 ± 95	3248 ± 97
	$l^{2}$	0.086 ± 0.017	0.087 ± 0.019	0.087 ± 0.017	0.093 ± 0.018
	$h_{a}^{2}$	0.176 ± 0.016	0.181 ± 0.015	0.194 ± 0.014	0.196 ± 0.014
	$h_{d}^{2}$	0.046 ± 0.009	0.049 ± 0.009		
	$h_{i}^{2}$	0.014 ± 0.005		0.019 ± 0.004	
	$e^{2}$	0.678 ± 0.022	0.684 ± 0.022	0.700 ± 0.021	0.711 ± 0.021
BF	$\sigma_{l}^{2}$	0.059 ± 0.019	0.066 ± 0.016	0.069 ± 0.018	0.070 ± 0.019
	$\sigma_{a}^{2}$	0.341 ± 0.018	0.342 ± 0.018	0.350 ± 0.018	0.352 ± 0.017
	$\sigma_{d}^{2}$	0.045 ± 0.012	0.046 ± 0.010		
	$\sigma_{i}^{2}$	0.015 ± 0.005		0.015 ± 0.005	
	$\sigma_{e}^{2}$	0.657 ± 0.023	0.664 ± 0.021	0.677 ± 0.021	0.689 ± 0.021
	$l^{2}$	0.053 ± 0.017	0.059 ± 0.017	0.062 ± 0.019	0.063 ± 0.018
	$h_{a}^{2}$	0.305 ± 0.014	0.306 ± 0.013	0.315 ± 0.013	0.317 ± 0.013
	$h_{d}^{2}$	0.040 ± 0.009	0.041 ± 0.009		
	$h_{i}^{2}$	0.013 ± 0.005		0.015 ± 0.005	
	$e^{2}$	0.588 ± 0.020	0.594 ± 0.021	0.608 ± 0.020	0.621 ± 0.020

DG, average daily gain; BF, backfat thickness; ADI, full model includes additive, dominance and imprinting effects; AD, model includes additive and dominance effects; AI, model includes additive and imprinting effects; A, model includes the additive effect

^a^Variance components ± posterior standard deviations ($\sigma_{l}^{2}$: litter effect, $\sigma_{a}^{2}$: additive effect, $\sigma_{d}^{2}$: dominance effect, $\sigma_{i}^{2}$: imprinting effect, $\sigma_{e}^{2}$: residual effect) and their proportions to the sum of variance components in the model ± posterior standard deviations ($l^{2}$: litter effect, $h_{a}^{2}$: additive genetic, $h_{d}^{2}$: dominance effect, $h_{i}^{2}$: imprinting effect, $e^{2}$: residual effect)

For models that included dominance effects, dominance accounted for approximately 4.6 (model ADI) and 4.9 % (model AD) of the phenotypic variance for DG and 4.0 (model ADI) and 4.1 % (model AD) for BF. The $2{\text{lnK}}$ values between models AD and A were equal to 36 for both DG and BF, which indicates very strong evidence for the presence of dominance effects. The estimated dominance variance was 27.1 % of the additive genetic variance when using model AD and which decreased to 25.6 % for model ADI for DG, and similarly decreased from 13.4 to 13.2 % for BF. These results show that dominance effects contribute to the genetic variation of DG and BF.

For the models that included imprinting effects, imprinting accounted for approximately 1.4 (model ADI) and 1.9 % (model AI) of phenotypic variance for DG and 1.3 (model ADI) and 1.5 % (model AI) for BF. Although the estimates of imprinting variances were small, the $2{\text{lnK}}$ values between models AI and A were equal to 5 and 11 for DG and BF, respectively, which indicates evidence of imprinting effects for DG and very strong evidence for BF. The estimated imprinting variance was 9.8 % of the additive genetic variance when using model AI and decreased to 8.2 % when using model ADI for DG, and similarly decreased from 4.7 to 4.4 % for BF. Regardless of the model used, imprinting variance was smaller than dominance variance. Across all models, both dominance and imprinting variances were generally stably estimated, and were more stable for BF than for DG.

Based on model ADI, estimated narrow-sense heritability was equal to 0.176 for DG and 0.305 for BF, and estimated broad-sense heritability was equal to 0.236 for DG and 0.359 for BF.

### Reliability of predictions

Reliabilities of predicted total genetic values obtained with the alternative models are in Table 2. For both DG and BF, model AD provided the highest reliability, followed by models ADI, A, and AI. Reliabilities of the predicted GTV across models ranged from 16.1 to 18.4 % for DG and from 30.1 to 31.4 % for BF. Compared with model A, model AD yielded a significantly more reliable prediction of GTV for DG and BF (*P* = 0.03 and *P* = 0.048, respectively). Although reliabilities obtained with model ADI were also higher than those obtained with model A for both traits, these differences were not statistically significant.

**Table 2:** Reliabilities of predictions for the validation animals for different models of analysis

Trait	Model	Reliability^a^	
		$r_{GTV}^{2}$	$r_{GBV}^{2}$
DG	A	0.166	0.166
	AD	0.184 (0.030)	0.167 (0.763)
	AI	0.161 (0.051)	0.165 (0.334)
	ADI	0.170 (0.534)	0.166 (0.567)
BF	A	0.307	0.307
	AD	0.314 (0.048)	0.313(0.048)
	AI	0.301 (0.053)	0.307 (0.983)
	ADI	0.308 (0.865)	0.312 (0.048)

DG, average daily gain; BF, backfat thickness, A, model includes the additive effect; AD, model includes additive and dominance effects; AI, model includes additive and imprinting effects; ADI, full model includes additive, dominance and imprinting effects

^a^Reliability of the predicted total genetic value $(r_{GTV}^{2} )$, reliability of the estimated additive genetic effect $(r_{GBV}^{2} )$ and *P* value (in parentheses if significantly higher than with the A model)

Reliabilities of GBV ranged from 16.5 (model AI) to 16.7 % (model AD) for DG and from 30.7 (models AI and A) to 31.3 % (model AD) for BF. For DG, reliabilities of GBV obtained with models that included dominance and/or imprinting effects were similar to those obtained with the basic additive model (Table 2). For BF, the model that included dominance effects (model AD) significantly (*P* = 0.048) improved the reliability of GBV compared to model A, but for the model that included imprinting effects, reliabilities of the predicted GBV were similar to those obtained with the basic additive model.

### Bias of predictions

Bias of the predictions was measured as the regression coefficients of $y_{c}$ on GTV or GBV (Table 3). Across the four models, regression coefficients were significantly different from 1, which indicated that the predictions were generally biased. However, regression coefficients for predictions obtained with models that included dominance effects were slightly closer to 1 than those obtained with the model that did not include dominance effects, which suggests that the latter slightly reduced the bias of genomic predictions. Regression coefficients of $y_{c}$ on GTV ranged from 0.747 (model AI) to 0.824 (model AD) for DG and from 0.722 (model AI) to 0.738 (model AD) for BF. Regression coefficients of $y_{c}$ on GBV ranged from 0.791 (model A) to 0.872 (model AD) for DG and from 0.732 (model A) to 0.764 (model AD) for BF. The least biased predictions of both GBV and GTV were obtained with model AD. Although model AI resulted in the most biased predictions of GTV, including imprinting effects into the models did not increase the bias of predictions of GBV. Predictions for DG were generally less biased than those for BF.

**Table 3:** Regression coefficients of corrected phenotypes (*y*_*c*_) on predictions for the validation animals for different models of analysis

Trait	Model	Regression coefficient^a^	
		Reg_GTV_	Reg_GBV_
DG	A	0.791 ± 0.082	0.791 ± 0.082
	AD	0.824 ± 0.081	0.872 ± 0.088
	AI	0.747 ± 0.082	0.802 ± 0.083
	ADI	0.796 ± 0.081	0.850 ± 0.090
BF	A	0.732 ± 0.041	0.732 ± 0.041
	AD	0.738 ± 0.040	0.764 ± 0.042
	AI	0.722 ± 0.041	0.733 ± 0.041
	ADI	0.724 ± 0.040	0.762 ± 0.042

DG, average daily gain; BF, backfat thickness; A, model includes the additive effect; AD, model includes additive and dominance effects; AI, model includes additive and imprinting effects; ADI, full model includes additive, dominance and imprinting effects

^a^Regression coefficients of the corrected phenotypes on predicted total genetic values (reg_GTV_) ± standard errors and regression coefficients of the corrected phenotypes on estimated breeding values (reg_GBV_) ± standard errors

Regarding the intercept, across the four models and for both DG and BF, the ratio of the intercept relative to the mean phenotypic value ranged from −0.03 to 0.01. The average ratios were equal to −0.02 (DG) and −0.01 (BF) for GTV and equal to −0.01 (DG) and 0.01 (BF) for GBV. The intercepts for GBV were slightly closer to 0 than those for GTV.

## Discussion

This study investigated the variance components of additive, dominance and imprinting effects, as well as the accuracy of genomic predictions for average daily gain and backfat thickness in Danish Duroc pigs. The estimates of dominance and imprinting variances indicated that these two effects contribute to the total genetic variance of DG and BF. For the two traits analyzed in this study, including dominance effects into the models resulted in more accurate and less biased predictions of GTV and GBV. However, the predictive ability was not significantly improved by including imprinting effects in the models.

### Genetic variances of average daily gain and backfat thickness

Based on our data, the estimates of dominance variances relative to additive genetic variances were equal to ~26 % for DG and 13 % for BF. Based on the Bayes factors, the evidence for dominance variance was very strong. Many previous studies have used pedigree information to estimate dominance variance [31, 32]. In a study on growth and reproduction traits in Yorkshire pigs [2], the estimates of dominance variance ranged from 2.2 % of the phenotypic variance for number of piglets born alive to 4.8 % for backfat at 104.5 kg and to 10.3 % for days to 104.5 kg, while dominance variance as a percent of additive variance ranged from 11 % for backfat to 78 % for 21-day litter weight. Another study on South African Duroc pigs reported dominance effects that were small and not statistically different from 0 for the traits analyzed [33]; estimates of dominance variance were equal to 3.8 % of phenotypic variance for number of piglets born alive, 1.0 % for interval between parities, and 1.5 % for 21-day litter weight, and the corresponding values for dominance variance as a percentage of additive variance were equal to 44.4, 56.8 and 14.8 %, respectively.

In our study, the effects of imprinting were smaller than the effects of dominance for both traits. The estimated imprinting variance as a percentage of additive genetic variance was equal to 8 % for DG and 4 % for BF. Based on the Bayes factors, evidence for the imprinting variance was positive for BF and very strong for DG. Previous studies investigated imprinting effects for pig growth traits based on pedigree information. In three purebred pig populations, paternal imprinting explained from 5 to 7 % of the phenotypic variance for backfat thickness and from 1 to 4 % for growth rate, while maternal imprinted explained 2 to 5 % for backfat thickness and 3 to 4 % for growth rate [9]. Analysis of a Large White population revealed a significant genetic imprinting variance for 19 of the 33 performance traits studied, with 5 to 19 % of the additive genetic variance being controlled by imprinted loci [4]. The *IGF2* gene has been reported to be expressed only from the paternal allele and has an effect on muscle mass and fat deposition in pigs [10], but in the population we analyzed, the SNP in the *IGF2* gene was fixed for the favorable allele. Imprinting effects have also been reported for other traits, e.g., for teat number in pigs, two imprinted QTL were found that explained 1.3 and 2.2 % of the phenotypic variance [7]. More recently, a study on litter size in two commercial pig populations detected a SNP with a significant imprinting effect that explained 1.6 % of the phenotypic variance, corresponding to approximately 15.5 % of the additive genetic variance [34].

Estimates of dominance and imprinting variances vary largely across studies. In our study, the estimates for the two traits analyzed, as a percentage of phenotypic variance, were very different, which may indicate different underlying genetic mechanisms, which is also supported by the estimated narrow-sense heritabilities of ~0.18 for DG and 0.31 for BF. The heritability estimate obtained for DG (0.20) is similar and that for BF (0.45) is slightly lower than the estimates reported in [9]. In addition, small datasets can cause large variation in the estimates. As shown here, the standard errors of dominance and imprinting variances were large. The relative standard errors for the estimated dominance and imprinting variances, defined as the standard error divided by the estimated variance, were approximately 2.3 to 6.2 times greater than those for the additive genetic variance (Table 1). In a previous study on DG [1], the relative standard error for the variance of dominance effects was 4.5 larger than that for additive genetic variance. For imprinting variance, this ratio ranged from 2 to 8 in a study on backfat thickness and growth rate in three purebred pig populations [9]. These results show that large datasets are necessary to obtain accurate estimates of dominance and imprinting variances.

In our study, when dominance and imprinting effects were included into the model, variances of the additive genetic effect, random litter effect and residuals decreased, which indicate that if dominance and imprinting effects are not included in a model, their variances will be distributed to variances of additive genetic, litter, and residual effects. Correspondingly, an increase in the estimate of broad-sense heritability was observed for model ADI compared to other models. Similarly, Su et al. [1] reported that when dominance and epistasis effects were included in the model to evaluate DG based on a dataset of Danish Duroc pigs born from 1996 to 2009, there was a decrease in the estimate of additive genetic, litter and residual variances, as well as an increase in the estimate of broad-sense heritability. In the study conducted by Su et al. [1], dominance effects accounted for 5.6 % of the phenotypic variance for DG, which was slightly more than the values of 4.6 to 4.9 % obtained here.

### Genomic prediction of average daily gain and backfat thickness

Previous studies showed that dominance and imprinting variances are quite large for some complex traits [2, 4, 5, 9]. Therefore, it was expected that models that include dominance and imprinting effects would increase the accuracy and reduce the bias of genomic predictions. In our study, reliabilities of the predicted total genetic value that were obtained with the model that included additive and dominance effects (model AD) were, however, only 1.8 and 0.7 % higher than those obtained with the basic additive model for DG and BF, respectively. In addition, the models that included dominance effects slightly decreased the bias of genomic prediction. The reliabilities of the predictions of BF were higher than the corresponding reliabilities of DG, regardless of whether the model included dominance and imprinting effects, which confirms the findings of a previous study [35]. The significant improvements in the reliabilities using dominance models for predicting DG and BF were consistent with the significant dominance variances observed. For DG, although the models that included a dominance effect significantly increased the reliability of predicted GTV, the reliability of GBV was little affected. However, for BF, the reliability of the predicted GBV was also significantly increased by including dominance effects. The increase in reliability of GTV predictions may be mainly due to the more reliable prediction of additive genetic effects because models that include dominance effects may decompose the genetic effects in a more appropriate manner.

However, the models that included imprinting effects did not show any obvious superiority over the models that ignored imprinting effects and even yielded a slightly lower reliability. Similar or smaller reliabilities for models with compared to without imprinting effects might be due to the small imprinting variance relative to the phenotypic variance and to the difficulty in predicting imprinting effects. In contrast, Nishio and Satoh [14] did show an improvement in the predictive ability of models that include imprinting effects in a simulation study, which may have been due to several reasons: (1) the proportion of imprinting variance was larger in the simulation study in [14] than in our study; actually, Nishio and Satoh [14] showed that the improvement in the accuracy decreased as the proportion of imprinting variance relative to the total genetic variance decreased; (2) a large difference in the genetic variance between males and females can also reduce the performance of the model with imprinting effects [14], the difference between paternal and maternal variances was also observed in our study; (3) the paternal and maternal alleles were unknown in our study; Nishio and Satoh [14] showed that the improvement in accuracy from the inclusion of imprinting effects was smaller when the paternal and maternal alleles were predicted.

The lack of superiority of the models with imprinting effects could also be due to the additive genetic, dominance and imprinting genetic effects not being clearly distinguished from each other based on our dataset. A related issue is that, in our study, additive, dominance and imprinting effects were assumed to be independent in the parameterization of the model. However, there are alternative models that can account for dependencies between such genetic effects, e.g., BayesD [36]. Finally, it is expected that the advantage of including dominance and imprinting genetic effects in the genomic prediction model will be larger when a larger dataset with stronger relationships between individuals is available, because these affect the accuracy of parameter estimates.

In a practical genetic evaluation system, using a model that includes additive, dominance and imprinting effects for genomic prediction may be prohibitive in different ways. First, models that include genetic effects with two or three components often require much more computational power, thus more powerful computers and more efficient algorithms will be required. In our study, we used a Linux server with an Intel(R) Xeon(R) central processing unit (CPU) X 5677 (Intel Corp., Santa Clara, CA) at 3.47 GHz, with a total RAM of 95 GB and it took 8 h per 10,000 MCMC samples to compute 8113 animals with 99,012 (33,004 × 3) SNP effects in the full model, including additive, dominance and imprinting effects. Second, the phasing of paternal and maternal alleles might also be a limitation if both parents are not genotyped because it is difficult to distinguish the origins of the two alleles at a locus without the genotypic information from the parents. Furthermore, in order to use more phenotypic information, de-regressed estimated breeding values (DEBV) may be used instead of individual records [37]. However, the use of DEBV would not be appropriate for a model that includes dominance and imprinting genetic effects, because the genetic interaction effects in the offspring are not related to those in the parents. In addition, some bias might be generated by the pre-correction of phenotypic records, or come from the fact that the genotyped animals were not chosen randomly as is the case in our study. To solve these limitations, the inclusion of dominance and imprinting effects into a single-step model [38–41] that conducts genomic prediction using both genotyped and non-genotyped animals simultaneously might be an ideal approach, but, to date, such a method has not been developed.

Imprinting has a different effect on the phenotype of individuals than on the phenotype of their offspring [42]. At the level of the individual, the effects of imprinting are the same for males and females, which means that the relationship between genotype and phenotype is independent of sex. However, the effects of the male and female parents on the performance of their offspring are different with imprinting and this, therefore, requires sex-dependent breeding values in relation to the performance of offspring. In this case, the narrow-sense heritability can also be defined as the sum of additive and imprinting variances divided by total phenotypic variance [19]. If imprinting is important, selection on sex-dependent estimates of breeding values may increase the performance in the next generation.

In our Bayesian analyses, different mixing proportions in the prior for SNP effects were evaluated to test the sensitivity of genomic prediction to mixing proportions (results not shown). Eight different scenarios setting $\pi_{1}$ = 0.889, $\pi_{2}$ = 0.1, $\pi_{3}$ = 0.01, and $\pi_{4}$ = 0.001 or $\pi_{1}$ = 0.690, $\pi_{2}$ = 0.25, $\pi_{3}$ = 0.05, and $\pi_{4}$ = 0.01 for additive, dominance, and imprinting effects, respectively, were tested. Results (not shown) showed that the mixing proportions only affected the reliability of predictions at the third decimal. Thus, the predictive ability was not sensitive to the choice of parameters if they were within a reasonable range (i.e., most SNPs having a small variance and a few SNPs having a large variance).

## Conclusions

Our results showed that dominance and genomic imprinting effects contribute to the genetic variation of BF and DG in Danish Duroc pigs. Genomic prediction models that include dominance effects can improve the accuracy and reduce the bias of genomic predictions.

## References

[1] Su G, Christensen OF, Ostersen T, Henryon M, Lund MS (2012). Estimating additive and non-additive genetic variances and predicting genetic merits using genome-wide dense single nucleotide polymorphism markers. PLoS One.

[2] Culbertson MS, Mabry JW, Misztal I, Gengler N, Bertrand JK, Varona L (1998). Estimation of dominance variance in purebred Yorkshire swine. J Anim Sci.

[3] Moore GE, Ishida M, Demetriou C, Al-Olabi L, Leon LJ, Thomas AC, Abu-Amero S (2015). The role and interaction of imprinted genes in human fetal growth. Philos Trans R Soc B.

[4] Neugebauer N, Luther H, Reinsch N (2010). Parent-of-origin effects cause genetic variation in pig performance traits. Animal.

[5] de Koning DJ, Rattink AP, Harlizius B, Groenen MAM, Brascamp EW, van Arendonk JAM (2001). Detection and characterization of quantitative trait loci for growth and reproduction traits in pigs. Livest Prod Sci.

[6] Quintanilla R, Milan D, Bidanel JP (2002). A further look at quantitative trait loci affecting growth and fatness in a cross between Meishan and Large White pig populations. Genet Sel Evol.

[7] Hirooka H, de Koning DJ, Harlizius B, van Arendonk JA, Rattink AP, Groenen MA (2001). A whole-genome scan for quantitative trait loci affecting teat number in pigs. J Anim Sci.

[8] Stella A, Stalder KJ, Saxton AM, Boettcher PJ (2003). Estimation of variances for gametic effects on litter size in Yorkshire and Landrace swine. J Anim Sci.

[9] de Vries AG, Kerr R, Tier B, Long T, Meuwissen TH (1994). Gametic imprinting effects on rate and composition of pig growth. Theor Appl Genet.

[10] Nezer C, Moreau L, Brouwers B, Coppieters W, Detilleux J, Hanset R (1999). An imprinted QTL with major effect on muscle mass and fat deposition maps to the IGF2 locus in pigs. Nat Genet.

[11] Meuwissen TH, Hayes BJ, Goddard ME (2001). Prediction of total genetic value using genome-wide dense marker maps. Genetics.

[12] Hayes B, Goddard M (2010). Genome-wide association and genomic selection in animal breeding. Genome.

[13] Lopes MS, Bastiaansen JW, Janss L, Knol EF, Bovenhuis H (2015). Estimation of additive, dominance, and imprinting genetic variance using genomic data. G3 (Bethesda).

[14] Nishio M, Satoh M (2015). Genomic best linear unbiased prediction method including imprinting effects for genomic evaluation. Genet Sel Evol.

[15] Browning BL, Browning SR (2009). A unified approach to genotype imputation and haplotype-phase inference for large data sets of trios and unrelated individuals. Am J Hum Genet.

[16] Groenen MA, Archibald AL, Uenishi H, Tuggle CK, Takeuchi Y, Rothschild MF (2012). Analyses of pig genomes provide insight into porcine demography and evolution. Nature.

[17] Sargolzaei M, Chesnais JP, Schenkel FS (2014). A new approach for efficient genotype imputation using information from relatives. BMC Genom.

[18] de Koning DJ, Bovenhuis H, van Arendonk JA (2002). On the detection of imprinted quantitative trait loci in experimental crosses of outbred species. Genetics.

[19] Hu Y, Rosa GJ, Gianola D (2015). A GWAS assessment of the contribution of genomic imprinting to the variation of body mass index in mice. BMC Genom.

[20] Hu Y, Rosa GJ, Gianola D (2016). Incorporating parent-of-origin effects in whole-genome prediction of complex traits. Genet Sel Evol.

[21] Gao H, Su G, Janss L, Zhang Y, Lund MS (2013). Model comparison on genomic predictions using high-density markers for different groups of bulls in the Nordic Holstein population. J Dairy Sci.

[22] Janss L. Bayz Online Manual. 2016. http://www.bayz.biz/.

[23] Plummer M, Best N, Cowles K, Vines K (2006). CODA: Convergence Diagnosis and Output Analysis for MCMC. R News..

[24] Su G, Christensen OF, Janss L, Lund MS (2014). Comparison of genomic predictions using genomic relationship matrices built with different weighting factors to account for locus-specific variances. J Dairy Sci.

[25] Goodman SN (1999). Toward evidence-based medical statistics. 1: the P value fallacy. Ann Intern Med.

[26] Goodman SN (1999). Toward evidence-based medical statistics. 2: the Bayes factor. Ann Intern Med.

[27] Kass R, Raftery A (1995). Bayes factors. J Am Stat Assoc.

[28] Janss L, Sigsgaard T, Sorensen D (2014). Whole-genome analyses of lung function, height and smoking. Ann Hum Genet.

[29] Dunn OJ, Clark V (1971). Comparison of tests of the equality of dependent correlation coefficients. J Am Stat Assoc.

[30] Revelle W. psych: procedures for personality and psychological research. 2015. https://cran.r-project.org/web/packages/psych/index.html.

[31] de Boer IJ, Hoeschele I (1993). Genetic evaluation methods for populations with dominance and inbreeding. Theor Appl Genet.

[32] Misztal I (1997). Estimation of variance components with large-scale dominance models. J Dairy Sci..

[33] Norris D, Varona L, Ngambi JW, Visser DP, Mbajiorgu CA, Voordewind SF (2010). Estimation of the additive and dominance variances in SA Duroc pigs. Livest Sci.

[34] Coster A, Madsen O, Heuven HC, Dibbits B, Groenen MA, van Arendonk JA (2012). The imprinted gene DIO3 is a candidate gene for litter size in pigs. PLoS One.

[35] Jiao S, Maltecca C, Gray KA, Cassady JP (2014). Feed intake, average daily gain, feed efficiency, and real-time ultrasound traits in Duroc pigs: I. Genetic parameter estimation and accuracy of genomic prediction. J Anim Sci.

[36] Wellmann R, Bennewitz J (2012). Bayesian models with dominance effects for genomic evaluation of quantitative traits. Genet Res (Camb)..

[37] Ostersen T, Christensen OF, Henryon M, Nielsen B, Su G, Madsen P (2011). Deregressed EBV as the response variable yield more reliable genomic predictions than traditional EBV in pure-bred pigs. Genet Sel Evol.

[38] Legarra A, Aguilar I, Misztal I (2009). A relationship matrix including full pedigree and genomic information. J Dairy Sci.

[39] Aguilar I, Misztal I, Johnson D, Legarra A, Tsuruta S, Lawlor T (2010). Hot topic: a unified approach to utilize phenotypic, full pedigree, and genomic information for genetic evaluation of Holstein final score. J Dairy Sci.

[40] Christensen OF, Madsen P, Nielsen B, Ostersen T, Su G (2012). Single-step methods for genomic evaluation in pigs. Animal.

[41] Guo X, Christensen OF, Ostersen T, Wang Y, Lund MS, Su G (2015). Improving genetic evaluation of litter size and piglet mortality for both genotyped and nongenotyped individuals using a single-step method. J Anim Sci.

[42] Barlow DP, Bartolomei MS (2014). Genomic imprinting in mammals. Cold Spring Harb Perspect Biol.

